# Chen’s penetrating-suture technique for pancreaticojejunostomy following pancreaticoduodenectomy

**DOI:** 10.1186/s12893-023-02054-y

**Published:** 2023-05-29

**Authors:** Lihong Zhang, Xuefeng Zhu, Yongsheng Zhu, Jianjun Huang, Lide Tao, Yijun Chen

**Affiliations:** 1 Department of Hepatobiliary Surgery, Affiliated Hospital of Yangzhou University, 368 Hanjiang Road, Yangzhou, 225012 Jiangsu Province China; 2 Department of Hepatobiliary Surgery, Taixing People’s Hospital of Yangzhou University, 1 Changzheng Road, Taixing, 225400 Jiangsu Province China

**Keywords:** Pancreaticoduodenectomy, Chen’s Penetrating-Suture technique, Postoperative pancreatic fistula

## Abstract

**Background:**

Postoperative pancreatic fistula (POPF) is the most serious complication and the main reason for morbidity and mortality after pancreaticoduodenectomy (PD). Currently, there exists no flawless pancreaticojejunal anastomosis approach. We presents a new approach called Chen’s penetrating-suture technique for pancreaticojejunostomy (PPJ), which involves end-to-side pancreaticojejunostomy by suture penetrating the full-thickness of the pancreas and jejunum, and evaluates its safety and efficacy.

**Methods:**

To assess this new approach, between May 2006 and July 2018, 193 consecutive patients who accepted the new Chen’s Penetrating-Suture technique after a PD were enrolled in this study. Postoperative morbidity and mortality were evaluated.

**Results:**

All cases recovered well after PD. The median operative time was 256 (range 208–352) min, with a median time of 12 (range 8–25) min for performing pancreaticojejunostomy. Postoperative morbidity was 19.7% (38/193) and mortality was zero. The POPF rate was 4.7% (9/193) for Grade A, 1.0% (2/193) for Grade B, and no Grade C cases and one urinary tract infection.

**Conclusion:**

PPJ is a simple, safe, and reliable technique with ideal postoperative clinical results.

## Introduction

Pancreaticoduodenectomy (PD) is a standard procedure for a variety of malignant and benign diseases of the pancreas and periampullary region [1]. Although the mortality rate is low in high-volume institutions [2], the overall morbidity rate is high in various institutions [3, 4]. Postoperative pancreatic fistula (POPF) remains the single most important cause of morbidity, which can lead to prolonged hospitalizations, the need for repeated surgical interventions, and increased mortality rates [5]. Currently, there are many approaches for reconstruction the Pancreatic Stump and jejunum, including invaginated or binding and duct-to-mucosa sutures pancreaticojejunostomy,etc. [6–8]. However, there is still no consensus on the best method to reduce POPF. According to the anatomic and healing characteristics of the pancreas, the change of the traditional concept and a new technique of pancreaticojejunostomy are needed.

In 2006, our group established the new approach, which was a technique of end-to-side pancreaticojejunostomy by suture penetrating the full-thickness of the pancreas and jejunum, and the preliminary results at that time were quite encouraging [9]. Over the past 13 years, this approach has been used in 193 patients who underwent a pancreatico-jejunostomy after PD. The results were encouraging and showed that the approach could be performed with safety, simplicity, and a low pancreatic fistula rate.

## Patients and methods

From May 2006 to July 2018, 193 patients with periampullary malignancies underwent PD by using PPJ at the department of Hepato-Pancreato-Biliary Surgery, Taixing People’s Hospital of Yangzhou University, China. Among the 193 patients, 118 were male and 75 were female. The patients’ ages ranged from 25 to 85 years (mean, 67.6 years). One hundred and eighty-nine patients were diagnosed with a malignant disease by postoperative pathology, including 98 cases with carcinoma of the pancreatic head, 72 cases with cancer of ampulla of Vater and the lower part of the common bile duct, and 19 cases with adenocarcinoma of the duodenum. Four other patients were diagnosed with benign lesions, including 2 cases with chronic pancreatitis and 2 cases with adenoma of the pancreatic head. Of the 193 patients, 35 (18.1%) cases were associated with diabetes mellitus and 28 (14.5%) cases were associated with the presence of non-dilated pancreatic duct (< 2 mm diameter) and soft pancreas. Informed consent was obtained from all the patients or their families.This study was approved by the Ethics Committee of Taixing People’s Hospital of Yangzhou University. The ethics approval reference number is: 2018-TXL03-S012.

So as to powerfully analyze the surgical outcomes of the approach, the definition of POPF in the ISGPF (the International Study Group on Pancreatic Fistula Definition) was used in this study [10]. POPF was defined as follows: Any measurable volume of drainage fluid (output through the drainage tube placed surgically,or percutaneous drainage tube placed subsequently) on or after postoperative day 3, with an amylase level greater than three times the upper normal serum value. Drain fluid may have a “sinister appearance” that may vary from a dark brown to greenish bilious fluid (provided the anastomosis is close to a bilioenteric anastomosis) to milky water to clear “spring water” that appeared like pancreatic juice. Relevant clinical manifestations may include abdominal pain and distension with impaired intestinal function, and delayed gastric emptying; Fever (> 38 ℃), serum leukocyte count greater than 10,000 cells / mm3, and C-reactive protein may also increase. Imaging may be useful by identifying erosion or migration of the drain into an enteric viscus and thus the need for drain withdrawal to allow healing of the site of erosion. Imaging may help to determine whether the drainage tube is eroding or migrating to the intestinal viscera, thereby determining the need to remove the drainage tube to allow healing of the site of eroding.

### Technique

Dr. Yijun Chen and his teammates performed all the operationgs. Lymphadenectomy was integrated during radical resection of the uncinate process. Wedge and/or segmental resection and venous reconstruction were performed when the portal vein or superior mesenteric vein were invaded.No pylorus-preserving procedures were performed. The pancreas was transected with at least a 2 cm surgical margin from the tumor using an electrotome. After the surgical specimen was delivered, our new technique for pancreaticojejunal anastomosis was performed as described below.

### Preparation of the pancreatic stump

Hemostasis of the pancreatic remnant was carefully performed with electrical coagulation or absorbable sutures. The location of the main pancreatic duct was identified with a probe and a 2- to 3-mm diameter, 5–7-cm long plastic catheter was inserted as a stent to prevent stenosis after placing the interlocking sutures.

### Preparation of the jejunum loop

An approximately 45-50 cm long loop of the proximal jejunum was selected and a Roux-en-Y reconstruction method was used [11]. The contra mesenteric wall of jejunum was incised with along the longitudinal axis full thickness. The incision diameter was generally 2.5 cm to allow passage of the pancreatic stent catheter.

### Pancreaticojejunal anastomosis

**Fig. 1:**
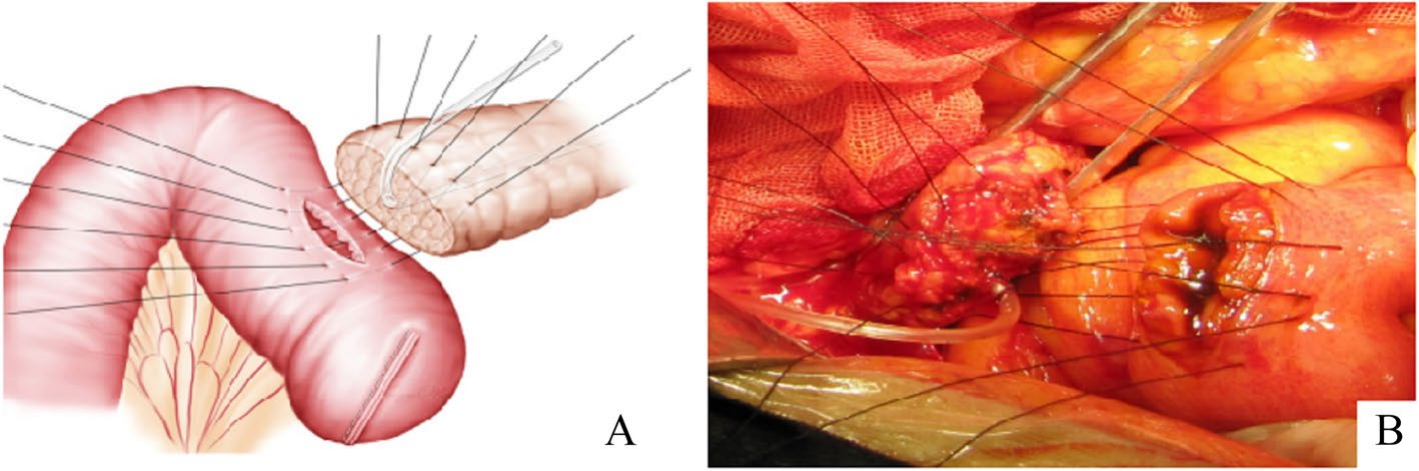
**A** and **B** The 4–0 Vicryl suture completely penetrates the pancreatic stump, then continuously penetrates from the posterior to the anterior wall of the jejunal loop. The same method was used to perform the subsequent 6–8 sutures. The stitch suturing the entire layer of the jejunal wall begins, from proximal to distal, approximately 2–3 cm to the resection margin of the loop. These sutures are preplaced approximately 8–10 mm from the cut edge of the pancreatic remnant and the jejunum, 5 mm between each other, and they are not tied until all 6–8 of the sutures have been placed. The first and last suture should be on the outer edge of the jejunum incision, so as to guarantee the pancreatic stump was completely covered with jejunum serosa

**Fig. 2:**
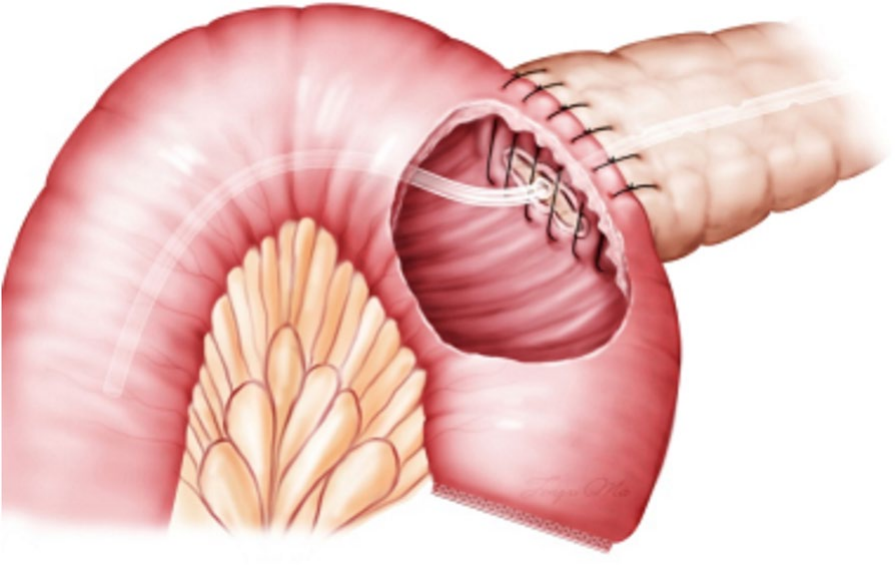
(diagram): Pancreatic stump was covered with jejunal wall and pancreatic duct catheter was put into the jejunum after anastomosis

**Fig. 3:**
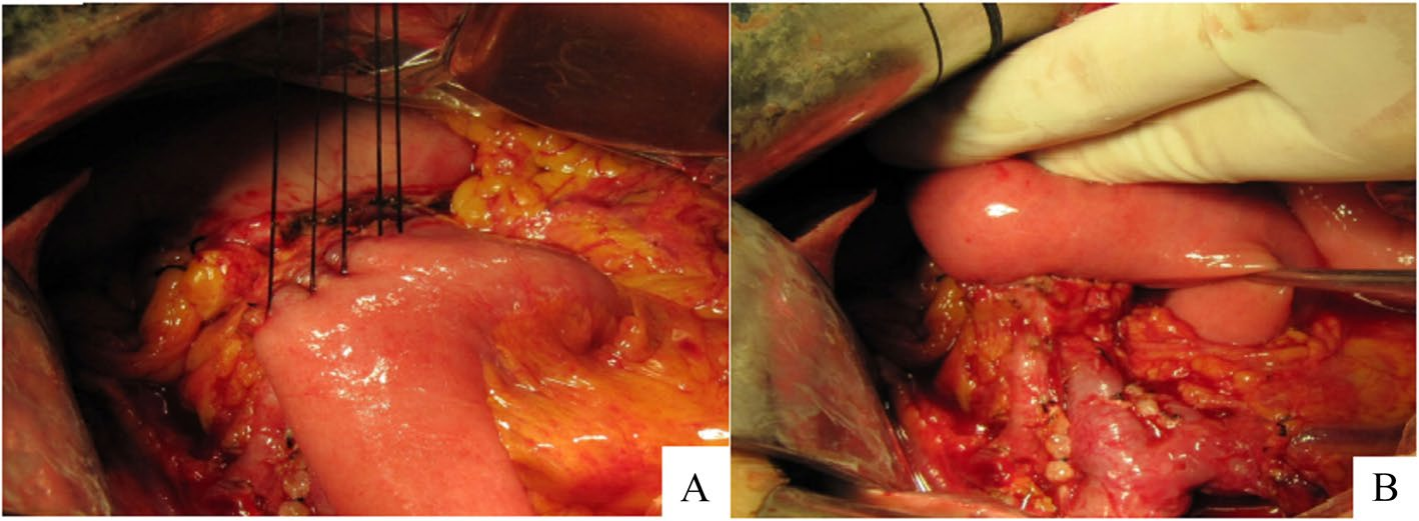
**A** The anterior wall of the anastomosis after being knotted. **B** The posterior wall of the anastomosis after being knotted

The anastomosis between the pancreatic remnant and the jejunal side wall begins. The first 4–0 Vicryl suture completely penetrates the pancreatic parenchyma from the anterior to posterior, and then continuously penetrates the posterior wall to the anterior wall of the jejunum loop. Subsequently, the first suture is completed and withdrawn after penetrating the entire pancreatic parenchyma and the full-thickness jejunum wall. Then, the same method was used to perform the second, third suture and until the completion of all of 6–8 sutures.The stitch spacing is 5 mm, the margin is 8-10 mm and they are not tied until all 6–8 of the sutures have been placed (Fig. 1). When penetrating the pancreatic parenchyma, it is essential to avoid the damage of the main pancreatic duct. After putting the pancreatic catheter to the jejunum, the knot was tightened slowly to ensure the jejunum incision and the pancreatic remnant smoothly matched and the jejunal wall varus (Figs. 2 and 3). Additional sutures were performed between the seromuscular layers of the anterior and posterior walls of jejunum loop and the pancreatic renmant to strengthen and achieve the pancreas-jejunum anastomosis.

### Other anastomosis

Further reconstruction of the continuity of the digestive tract includes end-to-side cholangiojejunostomy and end-to-side gastrojejunostomy. The gastrojejunostomy was carried out behind the transverse colon. A nasogastric tube with multi-lateral hole was placed in the input loop in all patients. After surgical reconstruction during PD, two drains were placed in the vicinity of the pancreaticojejunostomy and the cholangiojejunostomy without suction. The drainage tube remained intact 7–9 days after operation. The appearance and amount of drainage fluid were recorded daily, and drainage samples was collected on days 1–3 and 7 to test amylase level. For suspected clinical POPF, CT scan was performed to evaluate the situation of intraperitoneal pancreatic leakage and help to determine the next treatment. All patients took octreotide for 3 days after operation. The amylase level in the drainage fluid is an indicator to decide when to stop octreotide. Parenteral nutrition was started on postoperative day 0 and continued until the patient tolerated oral feeding.

## Results

Among all 193 patients, 165 patients with hard pancreas and dilated pancreatic duct and 28 patients with soft pancreas and small pancreatic duct underwent Chen’s Penetrating-Suture technique.

The median operative time was 256 (range 208–352) min, with a median time of 12 (range 8–25) min for performing pancreaticojejunostomy.The median blood loss was 475 ml (range 200–1300), 24 patients needed transfusion, and the median blood transfusion for these patients was 400 ml (range 300–800). The average length of stay of 193 patients was 15 days (range, 11–32 days). Overall morbidity rate was 19.7% (38/193). There were no cases of upper gastrointestinal bleeding. Ten patients (5.2%) had delayed gastric emptying, and gastric decompression was performed by placing a nasogastric tube. Other clinical complications included ascites in 15 cases (7.8%), respiratory tract infection in 6 cases (3.1%), urinary tract infection in 3 cases (1.6%), and wound infection in 4 cases (2.1%). All these patients recovered completely and received non-surgical treatment. According to the international clinical grading system, 11 patients (5.7%) developed POPF. Of these patients, 9 (4.7%) developed grade A-type POPF and 2 (1.0%) developed grade B-type POPF. Among them, there were 4 (14.3%)grade A and 1 (3.6%)grade B POPF in patients with a soft pancreas, and 5(3.0%)grade A and 1 (0.6%)grade B POPF in patients with a hard pancreas, respectively. The incidence of POPF was higher in patients with a soft pancreas than that in patients with a hard pancreas(*P* = 0.003).All patients did not have grade C-type POPF. The study group also had no death documented within 30 days after surgery (Table 1). The patients who had grade A-type POPF were treated conservatively with oral diet without additional interventions. For the patients with grade B POPF, it is very important to maintain the drainage unobstructed and prolong the use of inhibition of trypsin drug. If the Originally placed drainage tube was obstructed, and/or an abdominal abscess was developed, an additional interventional drainage procedure was needed. The nasogastric tube also helped obtain favorable clinical results. Other operation complications were cured by correlated conservative treatment. No anastomotic hemorrhage cases, no reoperation and no death cases (Table 1).

**Table 1:** The Data for Background Characteristics, Surgery Outcome, and Postoperative Complications

Background characteristics		
Gender, Male/Female	118/75	
Age, years	67.6 (25–85)	
Disease, n	193	
Carcinoma of the pancreatic head, n (%)	98 (50.8%)	
Cancer of ampulla of Vater, n (%)	34 (17.6%)	
Cancer of the lower part of the common bile duct, n (%)	38 (19.7%)	
Adenocarcinoma of the duodenum, n (%)	19 (9.8%)	
Benign diseases of the pancreas, n (%)	4 (2.1%)	
Outcome of operation		
Operation time, min		226 (162–352)
Pancreaticojejunostomy time, min		12 (8–25)
Operative blood loss, ml		475(200–1300)
Need transfusion, n (%)		24 (12.4%)
Blood transfusion, ml		400(300–800)
Hospital stay, day		15 (11–32)
Postoperative complications		
Morbidity rate, n (%)	38 (19.7%)	
Pulmonary infection, n (%)	6 (3.1%)	
Wound infection, n (%)	4 (2.1%)	
Urinary tract infection, n (%)	3 (1.6%)	
Ascites, n (%)	15 (7.8%)	
Delayed gastric emptying, n (%)	10 (5.2%)	
POPF, n (%)	11 (5.7%)	
Grade A, n (%)	9 (4.7%)	
Grade B, n (%)	2(1.0%)	
Grade C, n (%)	0	
Re-operation, n	0	
Mortality, n	0	

## Discussion

In general, pancreatectomy is considered a highly traumatic procedure with a mortality rate of about 5% [4, 12]. The incidence of POPF has varied from 2.53% to 31% after PD, thereby having a decisive influence on postoperative outcomes [13–16]. Grade C pancreatic fistula associated with pancreaticojejunostomy failure is a common and serious complication, and has become the leading cause of death in patients after surgery. As reported in the literature [17–19], POPF have many risk factors, such as patient’s age, level of jaundice, operation time, blood loss, anastomotic type, drain management, pancreatic texture, pancreatic duct size, original pathology, and others. Among these factors, pancreatic injury is a basic one. When the needle penetrates the parenchyma, the damage of cutting tear and tissue ischemia of the pancreatic stump occurs because of the soft and brittle pancreatic texture. The pancreatic stump injury can directly or indirectly cause pancreaticojejunostomy failure. Therefore, minimal damage to the pancreatic stump may improve the anastomosis and reduce Pancreaticojejunostomy failure. The traditional concept of pancreaticojejunostomy techniques considered that pancreaticojejun-ostomy was like gastrointestinal anastomosis and the pancreas was regarded as a hollow organ. Actually, the pancreas is a solid organ, pancreaticojejunostomy unlike gastrointestinal anastomosis, and the healing of pancreas and intestine, including pancreatic remnant and intestinal wall and pancreatic duct and intestinal mucosa also different from gastrointestinal anastomosis.

The traditional main methods to perform pancreaticojejunal anastomosis are the duct-to-mucosa anastomosis and the invagination technique [8, 20–23]. As guided by existing techniques, the pancreatic tissue was prone to be cutted and teared by the suture, and the quality of anastomosis cannot be guaranteed. The suture number and machinery and ischemic injury to the pancreas can lead pancreatic tissue damage and pancreatic leakage. As for cases of invagination anastomosis, pancreatic remnant was exposed in the jejunal lumen, the pancreatic parenchyma may be eroded, and then necrosis and hemorrhage may happen. As for cases of duct-to-mucosa anastomosis, succus pancreaticus of the pancreatic stump and liquefaction or necrosis tissue cannot be timely introduction of the jejunum, and these factors can lead to anastomotic failure. Difficult procedures and surgical experience deficiency can also cause anastomotic failure. In order to prevent the failure of pancreaticojejunostomy failure, several techniques have been used and evaluated [4, 15, 24–26], including the anastomotic site (the pancreaticojejunostomy and pancreatogastrostomy), direction of anastomosis (end-to-end and end-to-side), anastomosis layer (one layer or two layer or three layer anastomosis); suture method (interrupted suture, continuous suture, mattress suture, purse string suture and binding, etc.), suture material improvement (silk suture, Vicryl or non traumatic suture), and application of the pancreatic stent tube, etc.. All these improved methods cannot fundamentally prevent pancreaticojejunostomy failure. The main reasons is that the conception of anastomosis is not scientific [27]. As the traditional conception of anastomosis existing, the old problem was not completely resolved, and new problems emerged. For example, binding pancreatico-jejunostomy is used widely and it has greatly reduced the pancreatic leakage. However, this technique would be difficult to be performed when the size of the jejunum and pancreatic remnant cannot be precisely matched [28]. Too huge pancreatic remnant forced to be bound into jejunum will lead to anastomosis ischemia, eventually leading to fistula or bleeding. In addition, local pancreatic and intestinal fluid accumulation in the junction will increase anastomotic tension before intestinal peristalsis is restored, and sometimes the process of isolating the pancreatic remnant by at least 3 cm is quite time-consuming [29]. Therefore, the change of the traditional concept of pancreaticojejunostomy is essential.

According to the pancreatic anatomic structure and the pancreas- intestines healing characteristics, we changed the traditional concept of anastomosis, abandoned the technique of gastrointestinal anastomosis for pancreaticojejunostomy. We take the pancreas as a solid organ and anastomose it with jejunum. The suture completely penetrates the pancreatic stump and the whole layer of the jejuna wall. To complete the anastomosis only 6 to 8 sutures are needed. In this study, we introduced Chen’s Penetrating-Suture technique for end-to-side pancreaticojejunostomy. Of particular interest to this technique, only 9 patients (4.7%) developed grade a POPF and 2 patients (1.0%) developed grade B POPF. Of the 193 consecutive patients receiving PD, no patient developed grade C POPF. Therefore, by using this new surgical technique, pancreatic leakage related morbidity and mortality were largely avoided.

Compared with other studies, the incidence of POPF in this study was significantly reduced. Furthermore, the type of anastomosis mode has the following features: (1) This technique is simple, convenient, low technical requirements and easy to learn. (2) The anastomosis is reliable and this technique can be widely used. The traditional pancreatojejunostomy and modified ones only applied to the specific pancreatic stump. But how to choose anastmosis, there was no quantitative index. Surgeons chose different anastomotic mode entirely depended on their experience, abilities and preferences. If the choice is inappropriate, anastomotic failure may occur. Chen’s Penetrating-Suture technique is not affected by the texture of pancreas, the size of the pancreatic remnant and the pancreatic duct. It can be used for all remnant pancreases and can avoid anastomotic failure by the improper anastomotic mode selection. (3) The apposition suture of the pancreas and jejunum is neat and to facilitate healing. (4) Unlike invaginated pancreatojejunostomy, Chen’s Penetrating-Suture technique has a good effect of hemostasis for the pancreatic sections, and no anastomotic bleeding occur after operation. The jejunal wall covering pancreatic section is helpful for healing and hemostasis. There were no cases of anastomotic bleeding in this group of patients after operation.

## Conclusions

Evidence obtained from this study has shown Chen’s Penetrating-Suture technique following PD is easy to perform in technique, with evidently less time for PD compared with other procedures. Most importantly, this novel technique is safe, simple and reliable. However, larger prospective cohort series and prospective randomized studies are needed to further validate the outcome. At that time, this technique may be widely used in clinic.

## Data Availability

The datasets used and/or analyzed during the current study are available from the corresponding author on reasonable request.

## References

[1] Cameron JL, Riall TS, Coleman J (2006). One thousand consecutive pancreaticoduodenectomies. Ann Surg.

[2] Wolfgang CL, Pawlik TM (2013). Pancreaticoduodenectomy: time to change our approach?. Lancet Oncol.

[3] Topal B, Fieuws S, Aerts R (2013). Pancreaticojejunostomy versus pancreatico-gastrostomy reconstruction after pancreaticoduodenectomy for pancreatic or periampullary tumours: a multicentre randomised trial. Lancet Oncol.

[4] Narayanan S, Martin AN, Turrentine FE (2018). Mortality after pancreaticoduodenectomy: assessing early and late causes of patient death. J Surg Res.

[5] Complications SR, Pancreaticoduodenectomy A (2021). Surg Clin North Am.

[6] Senda Y, Shimizu Y, Natsume S, Ito S, Komori K, Abe T, Matsuo K, Sano T (2018). Randomized clinical trial of duct-to-mucosa versus invagination pancreaticojejunostomy after pancreatoduodenectomy. Br J Surg.

[7] Zhang X, Dong X, Liu P, Yan Y, Wei Y, Zechner D, Gong P, Vollmar B (2017). Binding versus Conventional Pancreaticojejunostomy in Preventing Postoperative Pancreatic Fistula : A Systematic Review and Meta-Analysis. Dig Surg..

[8] Xu J, Zhang B, Shi S, Qin Y, Ji S, Xu W, Liu J, Liu L, Liu C, Long J, Ni Q, Yu X (2015). Papillary-like main pancreatic duct invaginated pancreaticojejunostomy versus duct-to-mucosa pancreaticojejunostomy after pancreaticoduodenectomy: A prospective randomized trial. Surgery.

[9] Chen YJ, Zhu X, Huang JJ (2012). Penetrating-suture type of pancreaticojejunostomy. Chin J Hepatobiliary Surg.

[10] Bassi C, Dervenis C, Butturini G (2005). Postoperative pancreatic fistula: an international study group (ISGPF) definition. Surgery.

[11] Shah MM, Martin BM, Stetler JL, Patel AD, Davis SS, Sarmiento JM, Lin E (2017). Reconstruction options for pancreaticoduodenectomy in patients with prior roux-en-Y gastric bypass. J Laparoendosc Adv Surg Tech A.

[12] Mogal H, Vermilion SA, Dodson R, Hsu FC, Howerton R, Shen P, Clark CJ (2017). Modified frailty index predicts morbidity and mortality after pancreaticoduodenectomy. Ann Surg Oncol.

[13] E Nakeeb A, E Sorogy M, Hamed H, et al. Biliary leakage following pancreaticoduodenectomy: Prevalence, risk factors and management.Hepatobiliary Pancreat Dis Int. 2019;18(1):67–72 10.1016/j.hbpd.2018.10.005 30413347

[14] Karabicak I, Satoi S, Yanagimoto H (2017). Comparison of surgical outcomes of three different stump closure techniques during distal pancreatectomy. Pancreatology.

[15] Zhang L, Li Z, Wu X (2015). Sealing pancreaticojejunostomy in combination with duct parenchyma to mucosa seromuscular one-layer anastomosis: a novel technique to prevent pancreatic fistula after pancreaticoduodenectomy. J Am Coll Surg.

[16] Cao X, Wang X, Zhao B (2020). Correlation between Intraoperative fluid administration and outcomes of pancreatoduodenectomy. Gastroenterol Res Pract.

[17] McMillan MT, Vollmer CM (2014). Predictive factors for pancreatic fistula following pancreatectomy. Langenbeck's archives of surgery / Deutsche Gesellschaft fur Chirurgie.

[18] Hayashi H, Amaya K, Fujiwara Y (2021). Comparison of three fistula risk scores after pancreatoduodenectomy: a single-institution retrospective study. Asian J Surg.

[19] Harrell KN, Jajja MR, Postlewait LM (2020). Influence of margin histology on development of pancreatic fistula following pancreatoduodenectomy. J Surg Res..

[20] McMillan MT, Malleo G, Bassi C, S, et al. Pancreatic fistula risk for pancreatoduodenectomy: an international survey of surgeon perception. HPB (Oxford). 2017;19(6):515–524. 10.1016/j.hpb.2017.01.022 28202218

[21] Andrianello S, Marchegiani G, Malleo G (2020). Pancreaticojejunostomy with externalized stent vs pancreaticogastrostomy with externalized stent for patients with high-risk pancreatic anastomosis: a single-center, phase 3. Randomized Clinical Trial JAMA Surg.

[22] Xiang Y, Wu J, Lin C (2019). Pancreatic reconstruction techniques after pancreaticoduodenectomy: a review of the literature. Expert Rev Gastroenterol Hepatol..

[23] Sun X, Zhang Q, Zhang J (2016). Meta-analysis of invagination and duct-to-mucosa pancreaticojejunostomy after pancreaticoduodenectomy: an update. Int J Surg.

[24] Kim M, Shin WY, Lee KY (2017). An intuitive method of duct-to-mucosa pancreaticojejunostomy after pancreaticoduodenectomy: use of one-step circumferential interrupted sutures. Ann Hepatobiliary Pancreat Surg.

[25] Gomez T, Palomares A, Serradilla M, et al L. Reconstruction after pancreato-duodenectomy: Pancreatojejunostomy vs pancreatogastr-ostomy. World J Gastrointest Oncol. 2014;6:369–376. 10.4251/wjgo.v6.i9.369 PMC4163735 25232462

[26] Han HJ, Choi SB, Lee JS (2011). Reliability of continuous suture of pancreaticojejunostomy after pancreaticoduodenectomy. Hepato Gastro Enterol.

[27] Chen Y, Ke N, Tan C, Zhang H, Wang X, Mai G, Liu X (2015). Continuous versus interrupted suture techniques of pancreaticojejunostomy after pancreaticoduodenectomy. J Surg Res.

[28] Buc E, Flamein R, Golffier C (2010). Peng's binding pancreatico-jejunostomy after pancreaticoduodenectomy: a French prospective study. J Gastrointest Surg.

[29] Martino DI, M, Mora-Guzman I, Blanco-Traba YG, Díaz MC, Khurram MA, Martín-Pérez E.  (2019). Predictive Factors of Pancreatic Fistula After Pancreaticoduodenectomy and External Validation of Predictive Scores. Anticancer Res.

